# Adenosine A2A receptor activation reduces chondrocyte senescence

**DOI:** 10.1096/fj.202201212RR

**Published:** 2023-03-08

**Authors:** Benjamin Friedman, Ane Larranaga‐Vera, Cristina M. Castro, Carmen Corciulo, Piul Rabbani, Bruce N. Cronstein

**Affiliations:** ^1^ Division of Rheumatology New York University Grossman School of Medicine New York New York USA; ^2^ Division of Translational Medicine New York University Grossman School of Medicine New York New York USA; ^3^ Hansjorg Wyss Department of Plastic Surgery New York University Grossman School of Medicine New York New York USA

**Keywords:** adenosine, adenosine A2A receptor, aging, cartilage, chondrocytes, osteoarthritis, p53 variants, senescence

## Abstract

Osteoarthritis (OA) pathogenesis is associated with reduced chondrocyte homeostasis and increased levels of cartilage cellular senescence. Chondrosenescence is the development of cartilage senescence that increases with aging joints and disrupts chondrocyte homeostasis and is associated with OA. Adenosine A2A receptor (A2AR) activation in cartilage via intra‐articular injection of liposomal A2AR agonist, liposomal‐CGS21680, leads to cartilage regeneration in vivo and chondrocyte homeostasis. A2AR knockout mice develop early OA isolated chondrocytes demonstrate upregulated expression of cellular senescence and aging‐associated genes. Based on these observations, we hypothesized that A2AR activation would ameliorate cartilage senescence. We found that A2AR stimulation of chondrocytes reduced beta‐galactosidase staining and regulated levels and cell localization of common senescence mediators p21 and p16 in vitro in the human TC28a2 chondrocyte cell line. In vivo analysis similarly showed A2AR activation reduced nuclear p21 and p16 in obesity‐induced OA mice injected with liposomal‐CGS21680 and increased nuclear p21 and p16 in A2AR knockout mouse chondrocytes compared to wild‐type mice. A2AR agonism also increased activity of the chondrocyte Sirt1/AMPK energy‐sensing pathway by enhancing nuclear Sirt1 localization and upregulating T172‐phosphorylated (active) AMPK protein levels. Lastly, A2AR activation in TC28a2 and primary human chondrocytes reduced wild‐type p53 and concomitantly increased p53 alternative splicing leading to increase in an anti‐senescent p53 variant, Δ133p53α. The results reported here indicate that A2AR signaling promotes chondrocyte homeostasis in vitro and reduces OA cartilage development in vivo by reducing chondrocyte senescence.

## Graphical abstract

A2AR agonism induces cartilage regeneration in OA. A2AR ligation activates a pathway involving Sirt1/AMPK in human TC28a2 chondrocytes. There is decrease in expression of senescence genes p21, p16, and p53. In contrast, expression increased for anti‐senescent p53 splice variant Δ133p53⍺. A2AR ligation‐induced cytoplasmic localization of p21 and p16 and increased levels of the Δ133p53⍺ protein. Our study suggests that A2AR activation with intra‐articular injection of liposomal‐CGS21680 can reduce senescence and enhance cartilage regenerative potential to ameliorate OA.

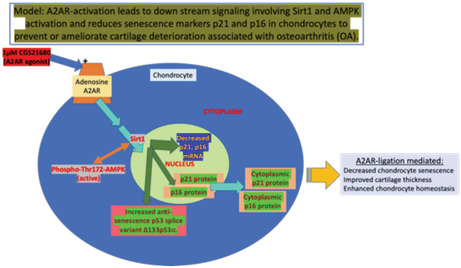

**Abbreviations**
A2AR: adenosine A2A receptor
A2ARKO: A2AR knockout

## 1 INTRODUCTION

Osteoarthritis (OA) is a common medical condition characterized by degeneration of articular cartilage and subchondral bone and increases with age affecting 25% of adults over age 65.^1^ Currently there are no targeted medical approaches for OA, which results in patient joint pain and decreased functional ability. Factors that predispose to the development of OA include advanced age, obesity, prior joint trauma, and genetic or anatomic factors.^2^ Aging and obesity are the most common risk factors, leading to cartilage destruction from physical forces on the joint and damage related to metabolic disease.^3^

OA is characterized by degraded articular cartilage that histologically displays reduced chondrocyte cellularity and increased degradation of surrounding extracellular matrix with a concomitant increase in matrix‐degrading enzymes such as MMPs.^2^ OA chondrocytes demonstrate reduction or dysfunction in common homeostatic mechanisms including autophagy, reactive oxygen species (ROS) regulation, and mitochondrial function.^4^

Adenosine is an endogenously produced nucleoside that activates a family of 4 G‐protein‐coupled receptors. The adenosine A2A receptor (A2AR) binds endogenous and exogenous adenosine to attenuate joint inflammation in vivo. Indeed, intraarticular injection of a liposomally packaged A2AR agonist prevents progression of and reverses obesity‐induced OA in mice and post‐traumatic OA (PTOA) in rats.^5^ Moreover, our laboratory has previously reported that A2AR signaling promotes autophagy, with a specific effect on mitophagy leading to improved mitochondrial function and reduction in chondrocyte oxidative stress.^6^

Endogenous A2AR signaling is also important for maintaining chondrocyte integrity and function; deletion of A2AR in mice leads to development of severe OA by early adulthood. In humans, loss of ecto‐5′nucleotidase, the enzyme responsible for hydrolyzing AMP to adenosine at the cell surface, also leads to the premature development of OA.^7^ Differential expression analysis of neonatal chondrocytes from A2ARKO mice revealed reduction in chondrocyte homeostatic pathways including autophagy, mitochondrial function (via mitophagy), and enhanced ROS production.^6^, ^8^, ^9^ Expression analysis of neonatal A2ARKO chondrocytes demonstrated increased gene expression in pathways related to cellular senescence, apoptosis, aging, and inflammation.^8^ We recently demonstrated that activation of the A2AR signaling pathway enhances chondrocyte autophagy in a FoxO‐dependent manner.^9^ This finding was consistent with prior data demonstrating that FoxO knockout mice had reduced cartilage homeostasis and early onset cartilage degeneration and OA.^10^

Cellular senescence is the state of stable proliferative cell cycle arrest in response to stimuli such as oxidative stress, pro‐inflammatory cytokine signaling, UV light exposure, and oncogenic stress. Senescent cells are detrimental to tissue homeostasis because they resist apoptosis and secrete several inflammatory mediators (the senescence‐associated secretory phenotype (SASP)). In OA cartilage there is increased chondrocyte senescence often associated with higher levels of pro‐senescence markers p53, p21, and p16, among others. On the other hand, higher activity of mediators such as Sirt1 and AMPK in chondrocytes seems to play a predominantly anti‐senescent role in cartilage and these proteins or their activity are decreased in OA.^11^, ^12^, ^13^, ^14^

Cellular senescence in articular chondrocytes causes tissue degeneration and removal of senescent chondrocytes improves joint histological appearance and function. Indeed, there are currently numerous senolytic agents undergoing clinical trials for the treatment of OA.^15^ Given that A2AR ligation increases chondrocyte homeostasis and leads to in vivo cartilage regeneration in rodent OA models,^16^ we determined whether A2AR agonist stimulation regulates cellular senescence associated with chondrocytes in osteoarthritic cartilage. In this work, we evaluated the effect of A2AR stimulation on senescence‐associated gene expression and protein levels, with a particular focus on cellular localization of senescence and anti‐senescence proteins in chondrocytes in vitro and in vivo. We also provide evidence for a novel anti‐senescence mechanism for A2AR; activation of A2ARs leads to differential post‐translational modification and increased alternative splicing of p53 to produce a truncated, anti‐senescent p53 variant that has opposing functions to p53.

## 2 METHODS

### 2.1 Materials

CGS21680 (A2AR agonist) and ZM241385 (A2AR antagonist) were obtained from TOCRIS (MI, USA). Paraformaldehyde (PFA) 16% was obtained from Electron Microscopy Sciences (PA, USA). RIPA buffer, EDTA, bovine serum albumin, Anti‐Rabbit IgG–FITC antibody, Anti‐Rabbit IgG–TRITC antibody, ethanol, glycerol, and adenosine were purchased from Sigma‐Aldrich (MO, USA). DMEM, penicillin–streptomycin, and fetal bovine serum were purchased from Life Technology (NY, USA). Antibodies (Primary antibody species—Rabbit, Rb; Mouse, Ms): Ms‐p53 (ab26), Rb‐p53 K382Ac (ab75754; K382Ac, acetylated lysine position 382), Rb‐p21/CDKN1A (ab109520; used for human cell western blotting and in vitro immunofluorescence), Rb‐p21/CDKN1a (ab188224; used for immunohistochemical staining on mouse sections in vivo), Rb‐p16/CDKN2A (ab108349; used for human cell western blotting and in vitro immunofluorescence), Rb‐p16/CDKN2A (ab189034; used for immunohistochemical staining on mouse sections in vivo), Ms‐Sirt1 (ab110304), Rb‐LKB1 (ab185734), Rb‐FOXO1A (ab 39670), and Ms‐nuclear matrix protein p84 (ab487) were all purchased from ABCAM (MA, USA). Ms‐β‐actin (A1978) was purchased from Sigma‐Aldrich (St. Louis, USA). Ms‐p53/DO‐11 (MCA1704) was purchased from Bio‐Rad (Hercules, CA, USA). Ms‐p53 (CST #2524), Rb‐AMPKα (CST #2532), Rb‐Phospho‐AMPKα (CST #2532) were each purchased from Cell Signaling Technologies (Danvers, MA, USA). Ms‐cGAS (sc‐515802), Ms‐dsDNA Marker (sc‐58749), Ms‐GAPDH (sc‐47724) were each purchased from Santa Cruz (CA, USA).

### 2.2 Animals

Mice utilized in this study are the subject of a separate report detailing the effect of liposomal adenosine and CGS21680 on reversal of OA in the obesity‐induced model of OA.^5^, ^9^, ^16^ Briefly, these mice were 12‐week‐old C57/Bl6 mice (Taconic Bioscience Laboratories, Hudson, NY, USA) fed a high‐fat diet (60% fat, Research Diets D12492i—Research Diets Inc., NJ, USA) for 12 weeks before liposomal intra‐articular injections (liposome alone, liposome attached to adenosine, liposome attached to A2AR agonist CGS21680). All protocols were approved by the New York University School of Medicine Institutional Animal Care and Use Committee.

### 2.3 Histology and immunohistochemistry

Cartilage section preparation is detailed elsewhere.^5^, ^9^, ^16^ Immunostaining was performed with specific primary antibodies at manufacturer‐specified dilutions overnight followed by washing and addition of secondary antibody using either goat anti‐rabbit IgG‐FITC and/or goat anti‐mouse‐TRITC (Sigma‐Aldrich) for an hour at 1:100 dilution ratio in phosphate‐buffered saline with 0.1% Tween 20 (PBST). Nuclei were counterstained with 4′,6‐diamidino‐2‐phenylindole (DAPI) mounting medium (ab104139, Abcam, MA, USA). Dilutions for the primary antibody incubations: Rb‐p21/CDKN1a (ab188224) at 1:1000 and Rb‐p16/CDKN2A (ab189034) at 1:200.

### 2.4 Human chondrocyte cell line and primary human chondrocyte culture

TC28α2 chondrocyte cells were maintained as described previously.^5^, ^9^, ^16^ Cells were treated with 1 μM of A2AR agonist CGS21680 for all experiments in this work for the allotted time as described in the text. In some experiments, cells were briefly pre‐treated for 5 min with A2AR agonist ZM241385 at a concentration of 1 μM prior to the addition of CGS21680 for all experiments in this work. Hydrogen peroxide (H_2_O_2_) was added in certain experiments simultaneously with CGS21680 at a fixed concentration of 100 μM for the two experiments assessing either in vitro beta‐galactosidase staining or p21 and p16 levels by immunofluorescence. A varying level of H_2_O_2_ (0, 50, or 100 μM) was used for a supplemental experiment in assessing p53 in vitro immunofluorescence. Beta‐galactosidase staining to detect senescence in TC28α2 cells was performed according to the manufacturer's protocol Senescence Detection Kit (ab65351). Primary human chondrocytes were obtained from reconstructive surgical samples isolated from discarded surgical samples as pre‐chondroblasts and subsequently differentiated into human chondrocytes based on established protocol.^6^, ^17^ When chondrocytes were fully differentiated, they were incubated with or without CGS21680 at 1 μM for 1 h for the protein extraction for western blot.

### 2.5 Protein extraction and western blotting

Protein from the TC28a2 human chondrocyte cell line or human primary chondrocytes cells was collected from total cell lysates followed by western blotting that was performed as detailed elsewhere.^5^, ^9^, ^16^ Protein was extracted from TC28a2 cells at approximately 80%–90% confluence for western blots. Approximately 30 μg total protein was loaded for each of the western blots in this work, which was determined by measuring protein concentration on cell lysates with a BCA assay kit (Thermo Scientific, Waltham, MA, USA) and calculated based on absorbance at 750 nm. Western blotting was performed by electrophoresing protein through 8%–16% polyacrylamide Mini‐PROTEAN pre‐cast protein gradient gels (Bio‐Rad, Hercules, CA, USA) followed by transfer of proteins to nitrocellulose membranes. Dilutions for the primary antibody incubations Tris‐Bu: Ms‐p53 (ab26) at 1:1000, Rb‐p53 K382Ac (ab75754) at 1:1000, Rb‐p21/CDKN1A (ab109520) at 1:000, Rb‐p16/CDKN2A (ab108349) at 1:2000, Ms‐nuclear matrix protein p84 (ab487) at 1:1000, Ms‐Sirt1 (ab110304) at 1:1000, Ms‐GAPDH (sc‐47724) at 1:200, Ms‐β‐actin (A1978) at 1:5000, Rb‐AMPKα (CST #2532) at 1:1000, Rb‐Phospho‐AMPKα (CST #2532) at 1:1000, Ms‐p53 (CST #2524) at 1:1000, and Ms‐p53/DO‐11 (MCA1704) at 1:1000.

### 2.6 Immunofluorescence

TC28α2 cells were plated in 8‐well chamber slides and fixed with 4% paraformaldehyde, permeabilization, blocking, primary and secondary antibody treatments, and fluorescent imaging were performed as detailed in prior work.^5^, ^9^, ^16^ The cells were about 70%–80% confluent prior to initiation of in vitro immunofluorescence experiments. Dilutions for the primary antibody incubations (PBST and 1% BSA): Ms‐p53 (ab26) at 1:500, Ms‐p53 (CST #2524) at 1:100, Rb‐p53 K382Ac (ab75754) at 1:1000, Rb‐p21/CDKN1A (ab109520) at 1:1000, Rb‐p16/CDKN2A (ab108349) at 1:100, Ms‐Sirt1 (ab110304) at 1:200, Rb‐LKB1 (ab185734) at 1:100, Rb‐FOXO1A (ab 39670) at 1:100, Ms‐cGAS (sc‐515802) at 1:100, Ms‐dsDNA Marker (sc‐58749) at 1:100, and Ms‐β‐actin (A1978) at 1:100.

### 2.7 mRNA extraction, reverse transcription, and real‐time PCR (RT‐PCR or qPCR)

Total RNA was extracted from TC28α2 cells using an RNeasy Mini Kit (Qiagen, Invitrogen) following the manufacturer's protocol. Reverse transcription and quantitative PCR were performed as described elsewhere with forward and reverse primer sets for each target gene or variant.^5^, ^9^, ^16^ Upon conversion to cDNA, real‐time PCR reactions were performed for relative quantification TP53/p53 (forward: 5′‐TAA CAG TTC CTG CAT GGG CGG C‐3′; reverse 5′‐AGG ACA GGC ACA AAC ACG CAC C‐3′), Δ133p53 (forward: 5′‐TCC TAC AGT ACT CCC CTG CC‐3′; reverse: 5′‐ACC ATC GCT ATC TGA GCA GC‐3′), CDKN1A/p21 (forward: 5′‐TGG AAC TTC GAC TTT GTC AC‐3′; reverse: 5′‐CAC ATG GTC TTC CTC TGC T‐3′), CDKN2A/p16 (forward: 5′‐GAA GGT CCC TCA GAC ATC CCC‐3′; reverse: 5′‐CCC TGT AGG ACC TTC GGT GAC‐3′), as compared to control GAPDH (forward: 5′‐GAC ATC AAG GTG AA‐3′; reverse: 5′‐TGT CAT ACC AGG AAA TGA GC‐3′). RT‐PCR was performed on a Stratagene Mx3005P (Agilent Technologies, Santa Clara, CA, USA) with Brilliant SYBR Green Kit QPCR Master Mix (Agilent Technologies), according to the manufacturer's protocol.

### 2.8 Statistical analysis

Statistical comparisons and significance were determined using unpaired Student's *T*‐tests and one or two‐way analysis of variance followed with post‐hoc testing via Tukey's test to provide a multiple comparison‐adjusted *p*‐value, as appropriate, using Prism 8 (GraphPad Software, Inc., La Jolla, CA, USA). The results are reported as mean ± SD with *p*‐values <.05 considered statistically significant. Statistical significance level tests in plots were denoted as *p < .05, **p < .01, ***p < .001, and ****p < .0001.

### 2.9 Study approval

All animal protocols were previously approved by the New York University School of Medicine Institutional Animal Care and Use Committee. All methods were performed in accordance with New York University Medical Center guidelines and regulations.

## 3 RESULTS

### 3.1 A2AR activation by CGS21680 decreases in vitro chondrocyte senescence which is blocked by a selective A2AR antagonist

**FIGURE 1:**
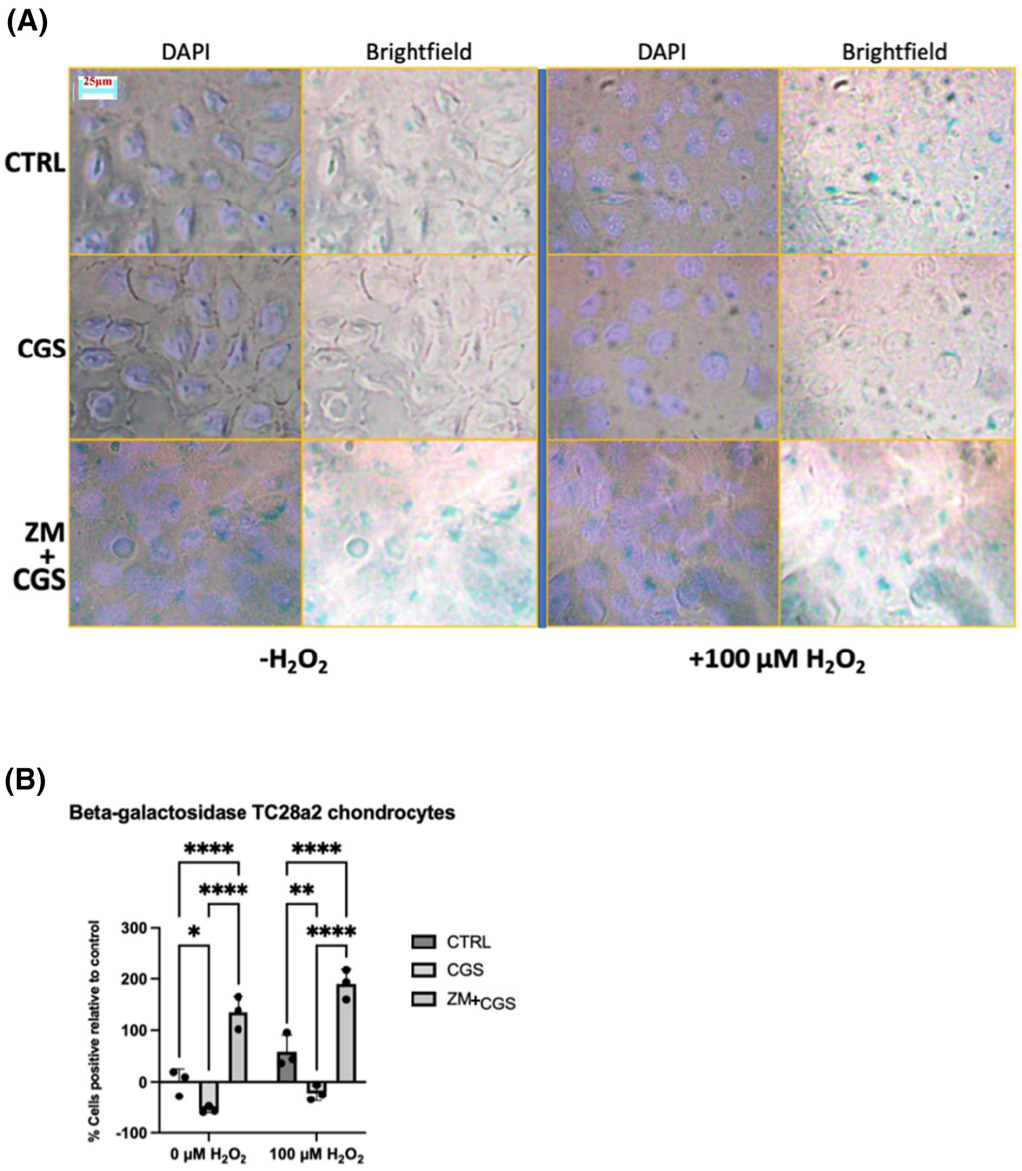
A2AR agonism reduces cellular senescence while A2AR inhibition increases senescence in TC28a2 chondrocytes. (A) This panel displays representative light microscope field images of cultured TC28a2 human chondrocytes treated under varying experimental conditions for 2 h with subsequent assay for beta‐galactosidase activity to estimate cell senescence. Experimental conditions for the TC28a2 chondrocytes included an untreated control (CTRL), cell treated with 1 μM CGS21680 (CGS) or without (CTRL) 1 μM A2AR agonist CGS21680 (CGS) or pre‐treated with 1 μM A2AR antagonist ZM241385 (ZM) in the presence or absence of 100 μM H_2_O_2_ followed by addition of 1 μM CGS21680 and cellular appearance was visualized by light microscopy. (B) Cell senescence was determined by percentage of cells staining positive for beta‐galactosidase after 2 h normalized to untreated cells. A grouped two‐way ANOVA with post‐hoc Tukey's tests for significance was performed using Prism comparing the treatments (CTRL, CGS, ZM) within either the 0 or 100 μM H_2_O_2_ groups. All the following comparisons are reported as percent difference of mean % beta‐galactosidase staining per group compared to control cells (± standard deviation with *n* = 3 per group). For 0 μM H_2_O_2_: CGS21680 versus CTRL −55 ± 6% versus 0 ± 25% (*p* = .048), ZM241385 versus CTRL 135 ± 32% versus 0 ± 25% (*p* < .0001), ZM241385 versus CGS21680 135 ± 32% versus −55 ± 6% (*p* < .0001). For 100 μM H_2_O_2_: CGS21680 versus CTRL −22 ± 15% versus 59 ± 32% (*p* = .005), ZM241385 versus CTRL 190 ± 29% versus 59 ± 32% (*p* < .0001), ZM241385 versus CGS21680 190 ± 29% versus −22 ± 15% (*p* < .0001). CTRL, untreated control chondrocytes; CGS, CGS21680; ZM, ZM241385; DAPI, 4′,6‐diamidino‐2‐phenylindole.

To assess the effect of A2AR ligation on chondrocyte senescence in vitro, we treated TC28a2 chondrocytes with or without 1 μM CGS21680 or antagonist pre‐treatment with 1 μM ZM241385 (followed by addition of 1 μM CGS21680) with or without oxidative stress (±100 μM H_2_O_2_) for 2 h and assessed chondrocyte senescence by beta‐galactosidase staining. We observed a significant decrease in the proportion of cells staining positive for beta‐galactosidase in the CGS21680 treated cells with or without H_2_O_2_, an effect that was completely reversed by A2AR antagonist ZM241385 (Figure 1).

We also examined the effect of A2AR agonism on nuclear structure and function as senescent cells have disrupted nuclear structure with increased micronuclei formation, decreased chromatin maintenance, decreased nuclear‐associated actin leasing to dysfunctional DNA repair and increased inflammation.^18^, ^19^, ^20^ A2AR activation reduces the presence of TC28a2 micronuclei, cytoplasmic dsDNA, and cytoplasmic localization of DNA sensor CGAS, effects that can be protective in the nucleus but promote senescence in the cytoplasm (Figures S1 and S2). We also found evidence that A2AR activation enhances nuclear membrane barrier function as measured by A2AR agonism‐associated decrease in TC28a2 cytoplasmic p84 (normally a nuclear marker) (Figure S3A,B). Furthermore, A2AR activation increases nuclear beta‐actin in TC28a2 cells (Figure S3C,D). This finding was supported by immunofluorescence in which A2AR agonism led to peri‐nuclear localization of actin compared to control cells with or without H_2_O_2_ treatment (Figure S3E). Collectively, these results indicate that A2AR activation decreases senescence‐like cellular changes in TC28a2 chondrocytes and that this diminishing effect on senescence by A2AR agonism is reversed by pre‐treatment of TC28a2 cells with A2AR antagonism. In fact, there were significantly increased numbers of senescence‐like cellular changes in the A2AR antagonist pre‐treated cells compared with control indicating that A2AR blockade not only eliminates the effect of CGS21680 but can enhance senescence above that observed in normal untreated cell culture.

### 3.2 A2AR stimulation decreases chondrocyte RNA expression of senescence markers p16 and p21 without altering total p16 or p21 protein abundance in vitro

**FIGURE 2:**
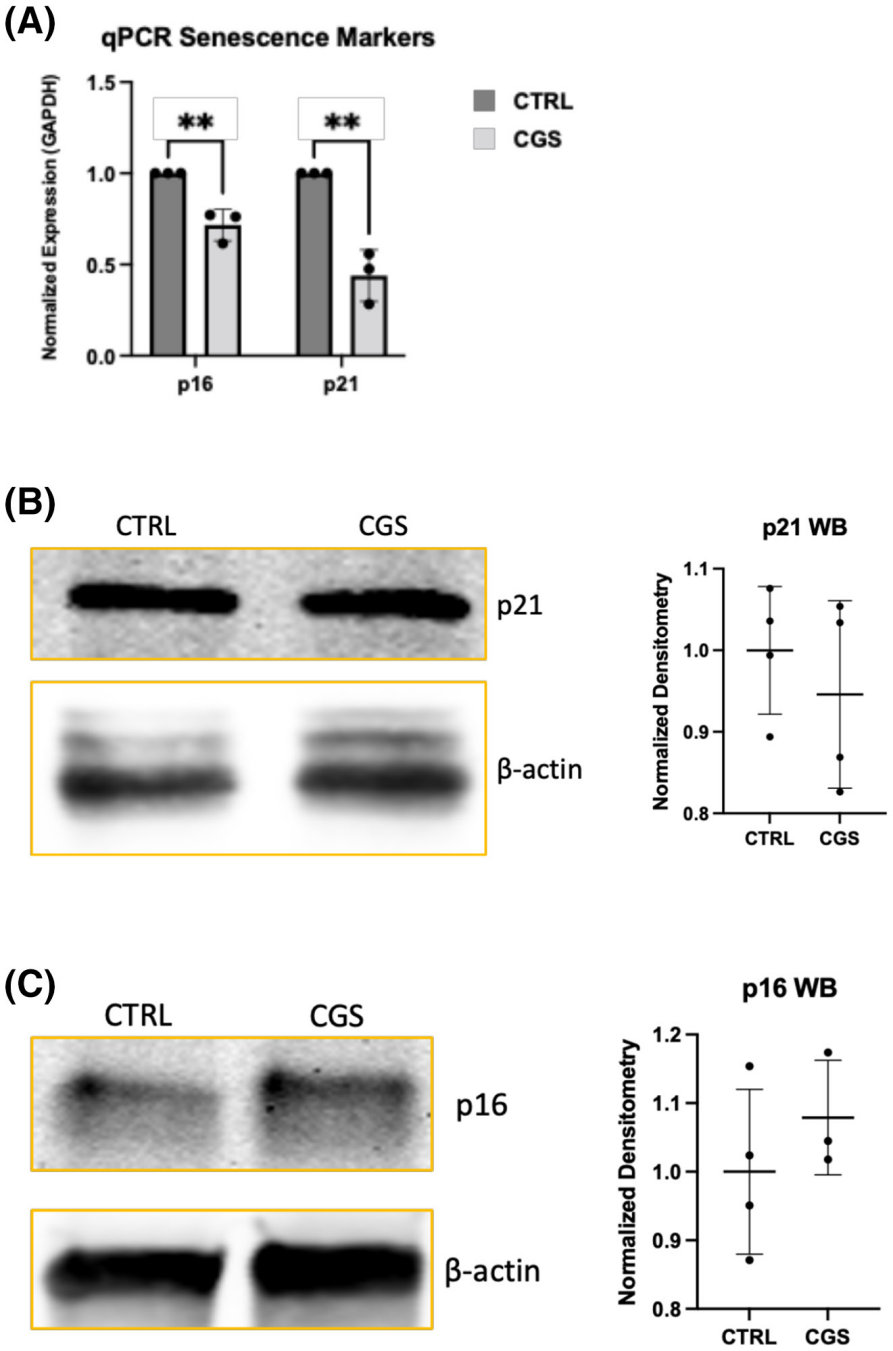
There is an A2AR‐associated decrease in RNA expression of senescence markers p16 and p21 without major change in overall total cellular protein levels. (A) A2AR activation in T/C‐28a2 chondrocytes treated ±1 μM CGS21680 led to significantly reduced RNA expression by *t*‐test for p16 (43 ± 14% reduction from control, *p* = .002, *n* = 3) and p21 (72 ± 9% reduction from control, *p* = .005, respectively, *n* = 3). Each individual qRT‐PCR experiment was performed in triplicate and 3 experiments were performed for both markers. Levels were normalized to GAPDH. (B) Western bot of cell protein extracts collected from TC28a2 cells treated ±1 μM CGS21680 for 1 h for p21 with graphical comparison without significant difference (*n* = 4 each). (C) Western blot of cell protein extracts collected from TC28a2 cells treated ±1 μM CGS21680 for 1 h for p16 with graphical comparison without significant difference (*n* = 4 each). Figures B and C are from total TC28a2 cell lysates and compared to beta‐Actin. CTRL, untreated control chondrocytes; CGS, CGS21680; p16, CDKN2A; p21, CDKN1A; qPCR, quantitative, real‐time polymerase chain reaction; WB, western blot; β‐Actin, beta‐Actin.

We subsequently determined the levels of major cell senescence markers in TC28a2 chondrocytes. Specifically, we measured p21/CDKN1A and p16/CDKN2A expression, both of which are elevated in senescent cartilage and other aged tissue.^4^, ^21^, ^22^, ^23^ We found that treatment with CGS21680 significantly decreases mRNA levels for both p16 and p21 (Figure 2A). However, we were surprised to find that there was no major change in cellular protein levels of either marker after CGS21680 treatment in TC28a2 cells (Figure 2B,C). Adjunctive to this in vitro analysis, we examined expression of general and/or OA‐associated SASP‐specific genes from our previously published RNA sequencing (RNAseq) data comparing expression changes in chondrocytes derived from A2AR knockout (A2ARKO) mice to controls. We noted statistically significant A2ARKO‐associated upregulation in gene expression for IL1α, IL‐6, PAI‐1/SERPINE1, CCL5, CCL7, M‐CSF/CSF1, G‐CSF/CSF3, CXCL1, CXCL2, CXCL3, CXCL5, PTGES, PTGES2, COX2/PTGS2, MMP13 (Figure S4). Based on the present experiments coupled with the in vivo gene expression analysis in A2AR knockout chondrocytes, we concluded that in vitro activation of A2AR signaling reduces the key senescence mediators p21 and p16 and A2AR null chondrocytes increase the production of many SASP factors in vivo.

### 3.3 A2AR signaling promotes cytoplasmic localization of p16 and p21 in chondrocytes in vitro

**FIGURE 3:**
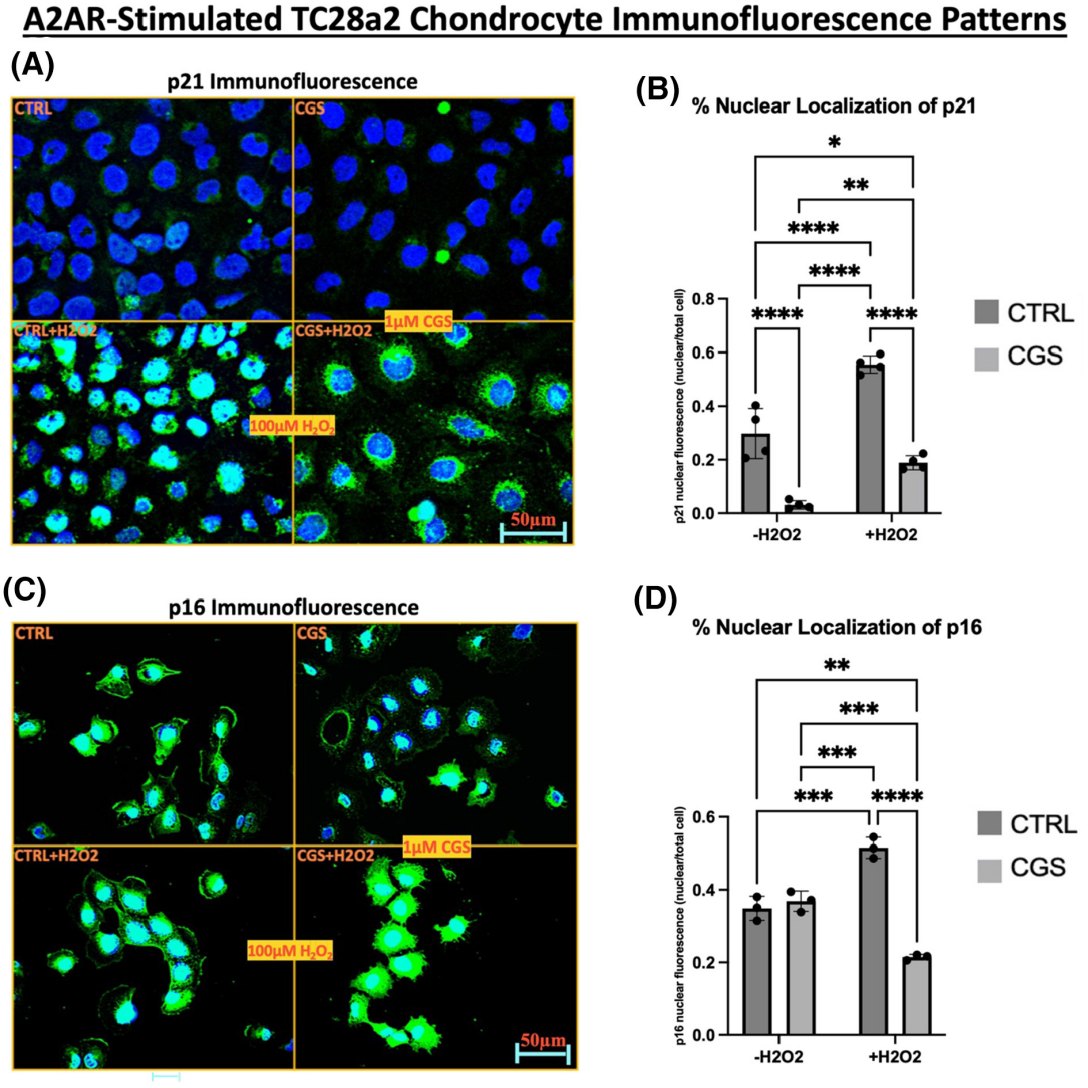
There is an A2AR‐mediated shift in localization of p21 and p16 by immunofluorescence in vitro in TC28a2 chondrocytes. (A) Cells were treated ±1 μM CGS21680 (right two panels) in the presence or absence of 100 μM H_2_O_2_ (bottom two panels) for 2 h and p21 localization was determined by immunofluorescence (green fluorescence). (B) Quantification of the relative nuclear intensity of p21 per cell (nuclear intensity divided by corresponding total cell intensity). This calculation was performed for each cell in a representative 40× high power field in separate experiments. Data identifying nuclear proportion of total cellular p21 fluorescence intensity are presented as means ± SD (*n* = 4). *p* values were calculated via two‐way ANOVA with post‐hoc Tukey's test. Data values (mean ± SD) obtained for the experimental conditions: control without H_2_O_2_, CTRL with H_2_O_2_, CGS21680 without H_2_O_2_, and CGS21680 without H_2_O_2_, were 0.30 ± 0.09, 0.56 ± 0.03, 0.03 ± 0.02, and 0.19 ± 0.03, respectively. (C) Cells were treated ±1 μM CGS21680 (right two panels with 1 μM CGS21680) presence or absence of 100 μM H_2_O_2_ (bottom two panels with 100 μM H_2_O_2_) for 2 h and p16 localization was determined by immunofluorescence (green fluorescence). These are representative images from three to four separate immunofluorescence experiments for each figure. Nuclei were stained with DAPI (blue). (D) Quantification of the relative nuclear intensity of p16 per cell (nuclear intensity divided by corresponding total cell intensity). This calculation was performed for each cell in a representative 40× high power field in separate experiments. Data identifying nuclear proportion of total cellular p21 fluorescence intensity are presented as means ± SD (*n* = 3). *p* values were calculated via two‐way ANOVA with post‐hoc Tukey's test. Data values (mean ± SD) obtained for the experimental conditions control without H_2_O_2_, CTRL with H_2_O_2_, CGS21680 without H_2_O_2_, and CGS21680 without H_2_O_2_, were 0.36 ± 0.05, 0.38 ± 0.01, 0.46 ± 0.03, and 0.22 ± 0.01, respectively. *p* values significance values are delineated in figures (B) and (D) as *p < .05, **p < .01, ***p < .001, ****p < .0001. CTRL, control; CGS, CGS21680.

Given A2AR activation did not alter protein levels, we next examined cellular localization of p21 and p16, because these proteins can have different effects on senescence based on their cellular localization.^24^, ^25^ We found that A2AR agonism of TC28a2 chondrocytes (1 μM CGS21680, 2 h) reduced nuclear and increased cytoplasmic p21 and p16 in treated‐dependently unique patterns in the presence or absence of oxidative stress (Figure 3A,B). Basal p21 fluorescence was faint in untreated and CGS‐treated TC28a2 chondrocytes, however, in the presence of H_2_O_2_, there was greater cytoplasmic p21 fluorescence in a punctate pattern in CGS‐treated chondrocytes. Basal p16 fluorescence in untreated cells demonstrated fluorescence in the nucleus and in certain regions in the vicinity of the cell periphery or membrane. CGS‐treated chondrocytes showed reduced nuclear p16 and increased cytoplasmic p16, indicating an A2AR stimulation involvement in mediating non‐nuclear p16 cell localization. Together, these qualitatively representative results indicate that A2AR ligation alters localization of p21 and p16 to the cytosolic compartment in vitro and this is enhanced by oxidative stress.

To quantitate each of the cell culture conditions (untreated control, +1 μM CGS21680, +100 μM H_2_O_2_, and +1μMCGS21680/+100 μM H_2_O_2_) we calculated the ratio of nuclear to total cellular fluorescence for p21 and p16. Nuclear fluorescence was determined using ImageJ to calculate the total fluorescent intensity of p21 or p16 colocalizing with the nucleus as represented by DAPI (blue). Proportional nuclear fluorescence was calculated by dividing the nucleus‐colocalized p21 or p16 fluorescence by the calculated total cellular intensity of either p21 or p16 for the corresponding entire cell. This assessment was performed by measuring fluorescent values in a representative 40× high power field three to four times in independent experiments for both p21 (*n* = 4) and p16 (*n* = 3). A two‐way ANOVA with post‐hoc Tukey tests to account for multiple comparisons demonstrated an A2AR‐associated significant decrease in proportion of nuclear p21 and p16 in the presence of oxidative stress (100 μM H_2_O_2_) and a significant decrease in nuclear p21 without H_2_O_2_ (Figure 3C,D). Together these results indicate enhanced A2AR signaling shifts p21 and p16 localization and appearance by immunostaining in TC28a2 chondrocytes in vitro.

### 3.4 Pharmacologic A2AR activation diminishes and A2AR knockout increases nuclear p21 and p16 localization in cartilage in vivo

**FIGURE 4:**
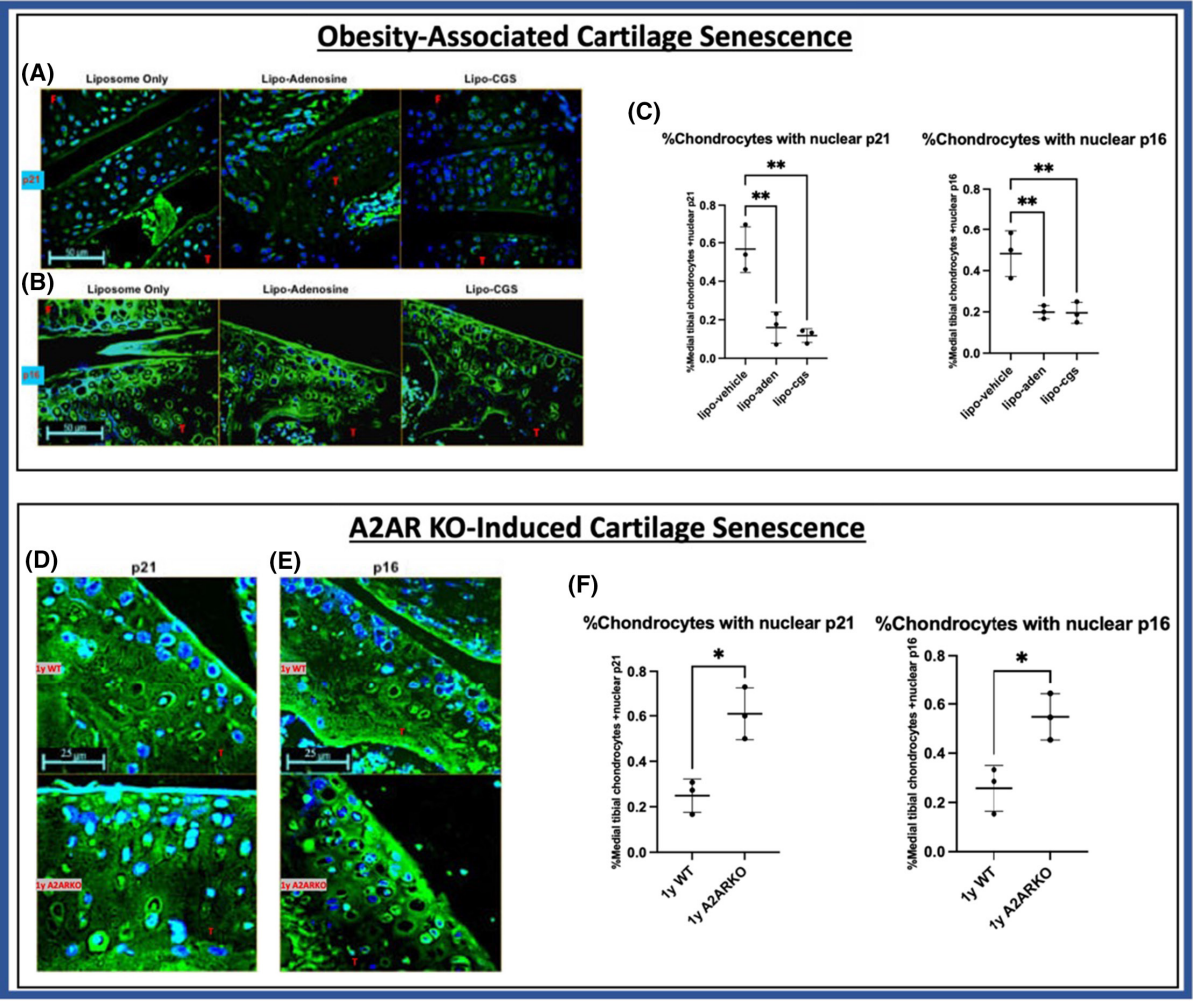
In vivo A2AR agonism reduces the presence of nuclear p21 and p16 and A2AR knockout mice have enhanced nuclear p21 and p16. Representative knee cartilage immunohistochemistry (IHC) for (A) p21 and (B) p16 levels in cartilage from 4‐month‐old obese OA mouse joints treated with intra‐articular injections with the empty liposome control, lipo‐adenosine, and lipo‐CGS21680 joints. Tibia (T), Femur (F). (C) For the obesity‐associated OA model treatment conditions, quantification of the in vivo chondrocyte nuclear fluorescence intensity was obtained for p21 and p16 levels in each chondrocyte identified in the medial tibial cartilage for each mouse. Individual nuclear p21 or p16 quantification was performed for each cell visible in the medial tibial cartilage. To obtain a proper comparative percentage of cells positive for nuclear p21 or p16, fluorescent thresholding using ImageJ was performed equivalently for all sections prior to counting chondrocytes in each section. Data are presented as means ± SD (*n* = 3). *p* values were calculated via one‐way ANOVA with post‐hoc Tukey's test for both markers evaluated, p21 and p16. For p21: data values (mean ± SD) for liposome alone, liposomal‐adenosine, and liposomal CGS were 57% ± 12%, 16% ± 8%, and 12% ± 4%, respectively. For p16: data values (mean ± SD) for liposome alone, liposomal‐adenosine, and liposomal CGS were 48% ± 11%, 20% ± 3%, and 20% ± 5%, respectively. Representative knee cartilage immunohistochemistry sections for (D) p21 and (E) p16 levels in cartilage obtained from 1‐year‐old mice that were wildtype or had knockouts in the A2AR gene (A2ARKO). (F) Quantification of percent medial tibial chondrocytes was performed as in part (C) of this figure. Data are presented as means ± SD (*n* = 3). *p* values were calculated using a Student's *t*‐test for each marker p21 and p16. For p21: values for mean ± SD for WT and A2ARKO were 25% ± 7% and 61% ± 11%, respectively. For p16: values for mean ± SD for WT and A2ARKO were 26% ± 9% and 55% ± 9%, respectively. *p* values are delineated in figures (C) and (F) as *p < .05, **p < .01, ***p < .001, ****p < .0001. WT, wildtype; A2ARKO, adenosine A2A receptor knockout, T, tibia; F, femur.

We next examined the effect of A2AR activation on p21 and p16 protein quantity and localization in vivo in a high‐fat diet (HFD)‐induced model of obesity‐associated OA treated with intra‐articular injections of liposomal‐CGS, liposomal‐adenosine, and an empty liposome control. In the obesity‐associated OA model of mice, there was increased nuclear localization of p21 and p16 in the chondrocytes in joints from mice injected with empty liposomes as compared to those treated with liposomal adenosine or liposomal CGS, findings consistent with the pattern of cellular localization observed in vitro (Figure 4A,B). Additionally, the effect of reduction of A2AR signaling on p21 and p16 was determined in vivo comparing 1 year old A2AR knockout mouse tibial cartilage sections to those from WT controls. IHC of these tissues reveals more cytoplasmic localization in wild‐type mice with greater nuclear localization of p16 and p21 in A2AR knockout mice (Figure 4C,D). These in vivo results are consistent with our findings in TC28a2 chondrocytes in vitro in which OA‐prone obese mouse joints treated with liposomal‐CGS21680 or liposomal‐adenosine joint injections have lower nuclear levels of p21 and p16 whereas a genetic knockout of the A2AR signaling pathway, which causes early OA, leads to higher nuclear p21 and p16.

### 3.5 A2AR ligation enhances in vitro chondrocyte protein levels of anti‐senescent mediators Sirt1 and phospho‐AMPK

**FIGURE 5:**
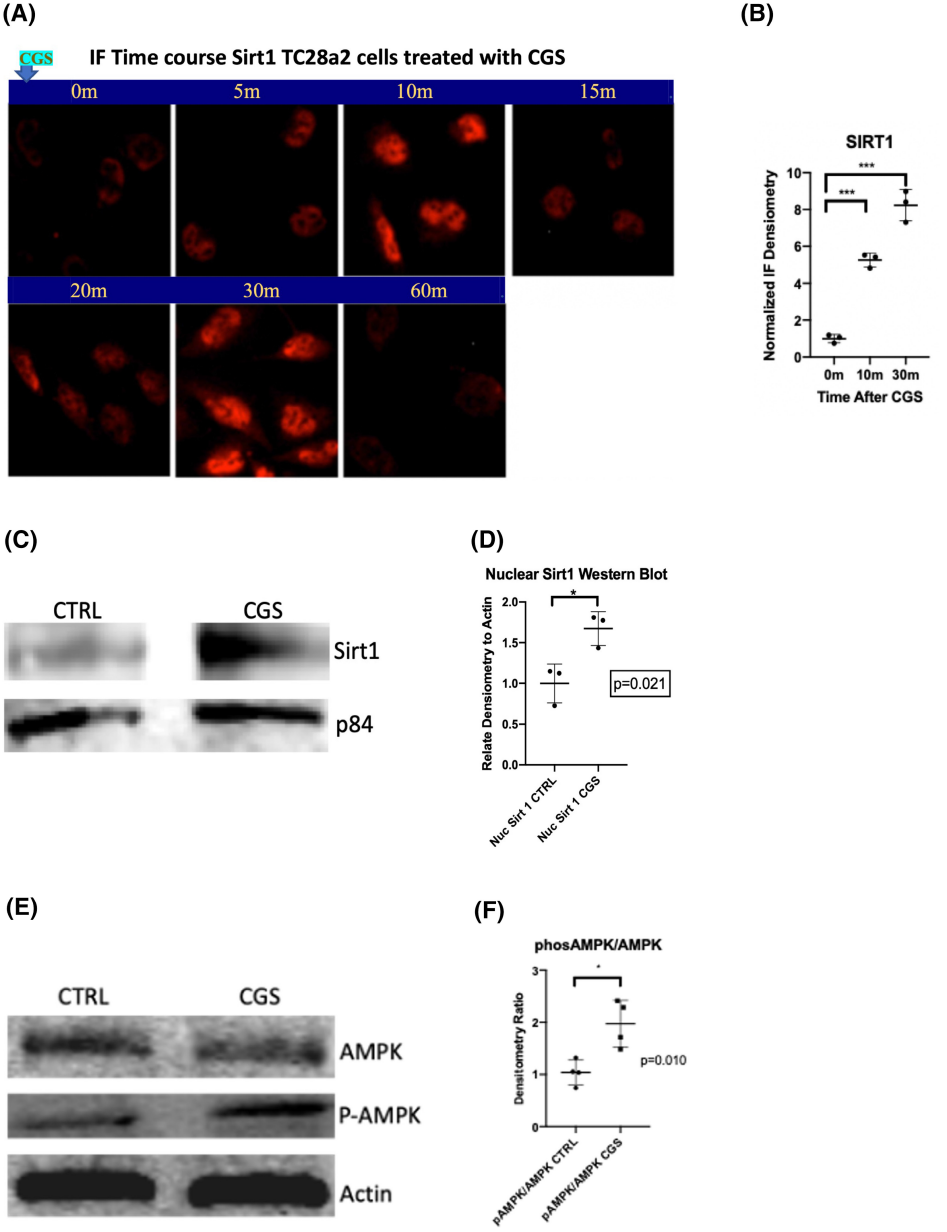
A2AR stimulation increases nuclear Sirt1and total cellular activated AMPK. (A) TC28a2 chondrocytes were treated with 1 μM CGS21680 for the displayed times from 0 to 60 min (Sirt1 displays red fluorescence here). Average Sirt1 fluorescence per cell was measured at baseline, 10 min after 1 μM CGS21680 treatment and 30 min after 1 μM CGS. (B) Normalized to control, average cellular Sirt1 fluorescence in cells from figure A was measured from representative 40× high power field in separate experiments for CGS21680 versus CTRL at 10 m (5.3 ± 0.4 vs. 1.0 ± 0.2, *p* = .001, *n* = 3, *t*‐test) and 30 m (8.2 ± 0.8 vs. 1.0 ± 0.2, *p* = .01, *n* = 3, *t*‐test). (C) Western blot showing TC28a2 Sirt1 immunostaining from the 30‐min nuclear fraction (compared to nuclear marker p84). (D) Comparison of protein levels from figure C western blot from 3 separate experiments, (1.7 ± 0.2 vs. 1.0 ± 0.2, *p* = .021, *n* = 3, *t*‐test). (E) Western blot for total AMPK and T172‐phospho‐AMPK (active) from TC28a2 cell lysates at 30 min after CGS21680 treatment. (F) Comparison for the ratio of phospho‐AMPK to total AMPK in each treatment group from figure E as determined from n of 4 per group, (2.0 ± 0.4 vs. 1.0 ± 0.2, *p* = .01, *n* = 4, *t*‐test). CTRL, untreated control chondrocytes; CGS, CGS21680; ZM, ZM241385, Sirt1, Sirtuin 1; m, minutes post CGS21680 exposure; p84, nuclear matrix protein p84 (nuclear protein loading control); AMPK, adenosine monophosphate kinase; P‐AMPK, Threonine (T172) phosphorylated AMPK (activated form); Actin (beta‐Actin, cell loading control).

To further understand how A2AR stimulation regulates cellular senescence we assessed quantity and activation of the anti‐senescent enzymes Sirt1 and AMPK, signaling molecules that have been implicated in cartilage homeostasis and shown to be protective against the development and progression of OA and numerous aging/degenerative conditions.^11^, ^12^, ^14^, ^21^, ^22^, ^26^, ^27^ We have previously reported that FoxO1 and FoxO3 are involved in A2AR signaling for enhanced autophagy in chondrocytes^9^ and it is known that both AMPK and Sirt1 can co‐regulate homeostasis through FoxO1/3 in cartilage post‐translationally by phosphorylation and deacetylation, respectively.^28^, ^29^, ^30^, ^31^ A2AR stimulation increases total cellular Sirt1 abundance in a bimodal fashion with peaks at 10 and 30 min after stimulation (Figure 5A–D) and induces nuclear colocalization of Sirt1 with FoxO1 which is apparent within 15–30 min after A2AR stimulation (Figure S5). A2AR signaling also leads to an increase in activated AMPK via increased phosphorylation (T172‐phospho AMPK, Figure 5E,F). Moreover, A2AR stimulation in TC28a2 chondrocytes leads to cytoplasmic translocation of the nuclear‐sequestered LKB1, which is a key upstream activating kinase for cytosolic AMPK (Figure S6). These findings are consistent with the hypothesis that A2AR stimulation leads to attenuation in chondrocyte senescence, in part, through activation of AMPK and Sirt1.

### 3.6 A2AR ligation stimulates an increase in an N‐terminal truncated splice variant of p53 which is anti‐senescent, anti‐aging in vitro

**FIGURE 6:**
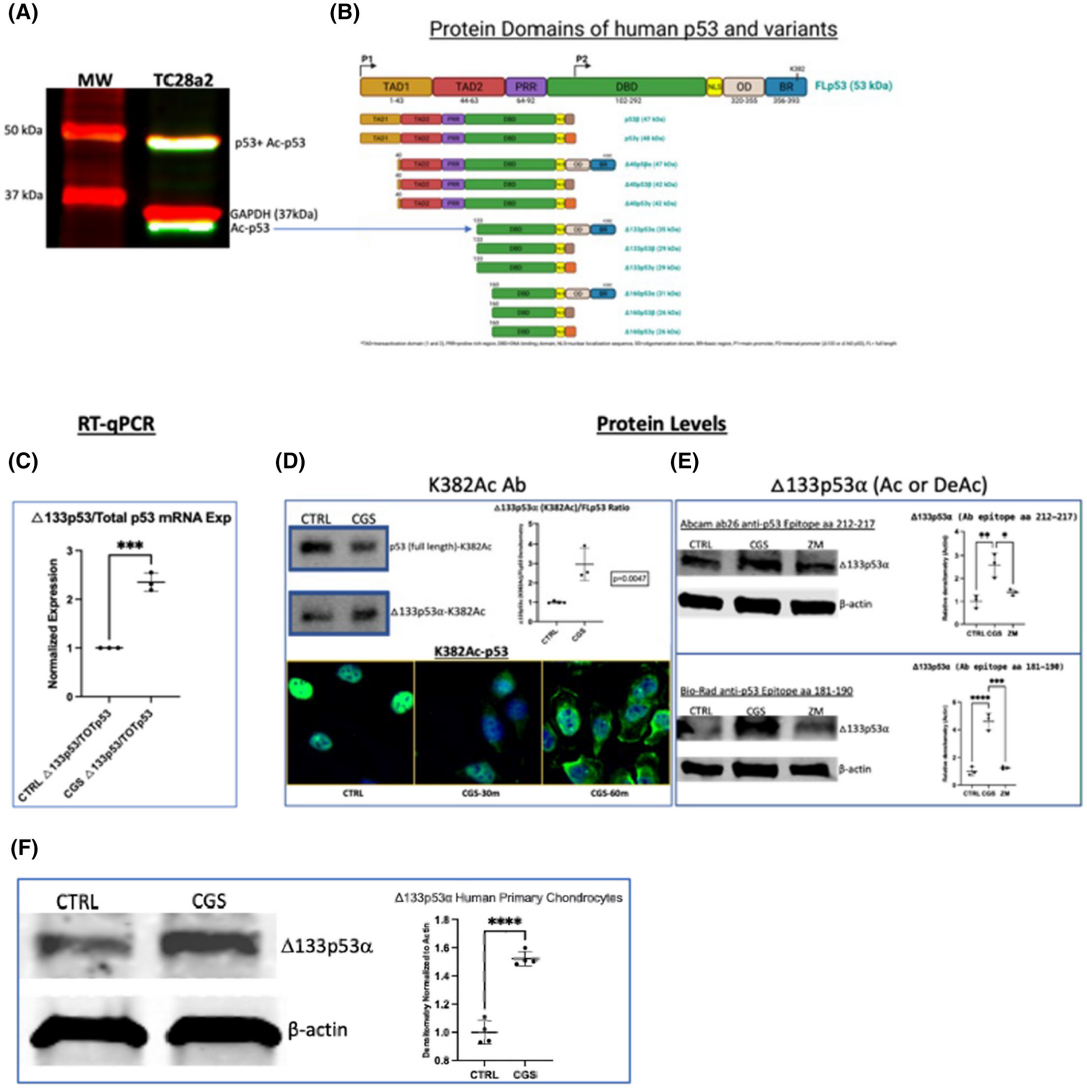
A2AR activation enhances the formation of an N‐terminally truncated anti‐senescence p53 variant, Δ133p53α. (A, B) These are the 12 currently known human p53 variants with the full‐length p53 (FLp53) at the top and the various N‐terminal and/or C‐terminal truncated variants below shown to the top right in figure B. In the top left figure (A) note the western blot image from a TC28a2 chondrocyte cell lysate we collected and immunostained for K382Ac‐p53 (green). This identified a larger protein of approximately the same MW as FLp53, also immunostained with an N‐terminus antibody corresponding to amino acids surrounding Ser‐20 in human p53 (red, with overlap appearing white/yellow). The K382Ac‐p53 antibody also recognized a smaller protein of around 35 kDa (green) as compared to known GAPDH with MW of approximately 36–37 kDa located just slightly above. Of the p53 variants, this most closely corresponds to Δ133p53α based on MW and the fact that K382 is only present in FLp53 and the α C‐terminal p53 variants. (C) RT‐qPCR from RNA collected from TC28a2 cells treated with or without 1 μM CGS21680 demonstrates relative increase in the RNA using primers that would recognize the Δ133p53 variants as compared to the primers for FLp53. (D) TC28a2 cells treated with or without 1 μM CGS21680 for an hour display a relative increase at the protein level using an antibody to K382Ac p53 of the Δ133p53α isoform to FLp53 (3.0 ± 0.8 vs. 1.0 ± 0.06, *p* = .005, *n* = 3–4). Below shows an immunofluorescence time course demonstrating change in p53 fluorescence as detected by this C‐terminal acetylated lysine 382 antibody in TC28a2 cells treated with CGS. (E) The WB findings from figure D were repeated using two alternate non‐acetylation‐dependent antibodies with mid‐p53 epitopes that recognize all known isoforms. Both antibodies demonstrate significantly increased levels of Δ133p53α in TC28a2 chondrocytes treated with CGS21680 that is reversed with pre‐treatment using A2AR antagonist ZM241385 (1 μM) followed by addition of agonist CGS21680 (labeled ZM241385 in the figure) (F) Western blot with antibody ab26 was performed on lysates from primary human chondrocytes treated with or without 1 μM CGS21680 for 1 h display a significant increase in the amount of Δ133p53α as assessed by western blotting of cell lysates treated with CGS21680 as compared to untreated chondrocytes (1.52 ± 0.051 vs. 1.00 ± 0.084, *p* < .0001, *n* = 4, *t*‐test). MW, molecular weight markers; CTRL, untreated control chondrocytes; CGS, CGS21680.

To further understand how A2AR stimulation regulates cellular senescence we determined the effect of A2AR stimulation with respect to p53, a central regulator of cellular senescence and suppressor of cellular proliferation, which is typically increased in cells damaged by oxidants and radiation as well as in cancer cells. Notably, it has been shown that senescence in chondrocytes from human OA patients and in cartilage from animal OA models is increased through a p53‐p21 pathway and that signaling through this pathway is reduced by Sirt1 deacetylation of p53.^22^, ^32^, ^33^ While we have previously demonstrated that A2AR ligation in TC28a2 chondrocytes significantly reduces total p53 protein levels,^9^ here we wished to determine the effect of A2AR pathway activation of Sirt1's ability to deacetylate p53 at lysine 382 using an antibody directed against Acetyl‐K382 p53. We did observe that A2AR stimulation led to a reduction in the levels of K382Ac p53 by western blot at its expected molecular weight but, surprisingly, leads to a concomitant A2AR‐associated increase in the quantity of both protein (Western Blot) and message (qPCR) for a 35 kD splice variant of p53, Δ133p53α, an N‐terminally truncated isoform that diminishes cellular senescence (Figure 6A–E). There are multiple p53 variants which include any combination of three N‐terminal truncations (Δ40p53, Δ133p53, Δ160p53) with three C‐terminal sequences (α, β, and γ, Figure 6A,B). There is a marked increase in the ratio of mRNA for Δ133p53α relative to full‐length p53 (Figure 6C). Figure 6D,E includes three western blots assessing protein levels of Δ133p53α in response to A2AR ligation. There is an increase in acetylated Δ133p53α that is acetylated at lysine 382 using an antibody to K382Ac‐p53 (Figure 6D) and the Western Blot shown in Figure 6E confirms the specificity of the antibodies used for the relevant isoforms. Lastly, we determined that the variant is indeed present in primary human chondrocytes, and it increases with A2AR activation, corroborating the findings for the TC28a2 chondrocytes (Figure 6F). These findings confirm that A2AR stimulation diminishes full‐length p53 and increases the amount of a p53 variant that has been found to be associated with reduced senescence, enhanced stem cell function, and improved longevity.

Further analysis of TC28a2 cells by IHC shows that Sirt1 plays an important role in localized deacetylation of p53 only in the A2AR‐stimulated chondrocytes. The K382 acetylated p53 is almost fully nuclear and the deacetylated p53 cytosolic and nuclear localization in CGS‐treated cells varies in an H_2_O_2_‐dependent manner in TC28a2 cells. Untreated TC28a2 chondrocytes display increasingly elevated deacetylated p53 fluorescence as oxidation level increases using both the mid‐ and N‐terminal p53 antibody (Figures S7 and S8) and is present in an increasingly diffuse, cytoplasmic, and disorganized pattern as H_2_O_2_ levels increase (the N‐terminal antibody signal remains mostly nuclear). The pattern of immunofluorescence localization of p53 and its isoforms is completely different in TC28a2 chondrocytes treated with the A2AR agonist. Firstly, A2AR stimulation induces increased levels of nuclear K382‐acetylation without N‐terminal antibody recognition, consistent with nuclear Δ133p53α. Importantly, in the presence of low‐mid range doses of H_2_O_2_ (50–100 μM), A2AR agonism leads to the formation of highly organized vesicular and globular nuclear structures that appear to be deacetylated at K382 and is not identified by the N‐terminal antibody. Overall, these findings are most consistent with the hypothesis that A2AR stimulation of TC28a2 chondrocytes augments protein abundance and localization of a beneficial anti‐senescent isoform of p53 and that there is cytosolic compartmentalization of deacetylated in the presence of oxidative stress.

## 4 DISCUSSION

A2AR stimulation promotes chondrocyte function and viability both in vitro and in vivo via multiple homeostatic pathways tied to cellular senescence. A2AR knockout mice, which cannot signal through the A2A receptor, and CD73 knockout mice, which have reduced levels of adenosine extracellularly in articular cartilage as a result of reduced conversion of ATP, both develop premature and spontaneous OA^16^ and intraarticular injection of liposomal adenosine or an A2AR agonist prevents OA progression and reverses, in part, established OA in a rat model of posttraumatic OA and a murine model of obesity‐induced OA.^5^, ^16^ Moreover, A2AR ligation stimulates in vitro and in vivo FoxO‐mediated autophagy, mitophagy, and improved mitochondrial function, thus leading to restoration of metabolic and redox homeostasis.^6^, ^9^ In rats with PTOA treated with intraarticular liposomal CGS21680 there was reduction of chondrocyte hypertrophy, cartilage catabolism, and chondrocyte apoptosis^5^ and, most interestingly, pathway analysis of differentially expressed genes in neonatal chondrocytes from A2AR deficient mice revealed that the most up‐regulated pathway was cellular senescence. Chondrocytes derived from neonatal A2AR knockout mice have a pattern of gene expression associated with senescence, apoptosis, and inflammation is consistent with the hypothesis that endogenous A2AR stimulation is required to maintain chondrocyte homeostasis. It is true that this RNAseq study of the A2AR pathway in cartilage examined neonatal chondrocytes, which may not be fully generalizable to OA chondrocyte gene expression changes. However, this study did recapitulate many of the changes in gene expression involved in the molecular foundations in developing early‐onset OA when cartilage is still grossly intact (as it still is in neonatal A2ARKO mice).

There may be numerous differences It is true that the RNAseq study using neonatal chondrocytes may be ha While studying A2ARKO‐associated changes Because of this evidence for A2AR activation in maintenance of cartilage homeostasis,^5^, ^6^, ^9^ we examined the direct effect of A2AR stimulation on senescence.

We found that A2AR stimulation inhibits spontaneous and oxidant‐mediated senescence in TC28a2 human chondrocytes. Treatment with an A2AR agonist reduced spontaneous and oxidant‐induced beta‐galactosidase activity and cell death in vitro. Moreover, A2AR stimulation reduced expression of message for p16 and p21 with an increase in cytoplasmic localization of these proteins. When studied in vivo in the articular chondrocytes of both A2AR knockout‐induced and obesity‐induced OA the increase in cytoplasmic p16 and p21 were recapitulated and treatment of obesity‐induced OA with intraarticular injections of liposomal adenosine or CGS21680 reversed this effect. Hence, our results indicated an adenosine A2AR‐mediated attenuation of chondrocyte senescence. This connection between A2AR agonism and reduced senescence has been observed in other cells. Activation of the A2AR has been implicated in attenuation of senescence as well as increased proliferation in mesenchymal stem cells and hematopoietic stem cells.^34^, ^35^

Senescent cells from many degenerative diseases express increased p16 and p21 and these proteins have been verified as markers for senescence in OA chondrocytes in humans and animal OA models. More specifically, prior work has indicated that p21, downstream of p53 in senescence, is directly involved in the transition from normal to senescent chondrocytes in OA whereas p16 is involved in the maintenance of senescence in degenerative cartilage.^4^, ^15^, ^36^ It is also important to note that p16, p21, as well as beta‐galactosidase function can be transiently elevated in physiologic situations involved in development or tissue repair (wound healing, for example).^37^, ^38^ Interestingly, reversibly elevated p16 and beta‐galactosidase staining were observed in functional, non‐senescent macrophages during normal physiological immune responses and this change was observed even in p53‐null cells.^39^ This finding in macrophages suggests that elevation in p16 without a concomitant increase in p53 is more likely involved in physiological processes reliant on reversible senescence.^39^ We previously demonstrated diminished p53 downstream of A2AR activation, suggesting that the decreased RNA expression of p16/p21 without change in protein could be transient within hours of A2AR stimulation.^9^

Recent reports have indicated the importance of maintaining functional, bidirectional nucleocytoplasmic trafficking of p16 and p21 in response to extrinsic and intrinsic stimuli to avoid cell senescence.^20^ While the exact mechanism by which A2AR‐mediated changes in localization for p16 and p21 needs to be further evaluated. Potential explanations for A2AR‐stimulated changes in p16 and p21 localization include increased autophagy as is seen in retinal pigmented epithelial cells (for p16)^25^ and threonine 145 phosphorylation of p21 by activated AKT. p16, which has no known nuclear export signal (NES), can localize to autophagosomes in the cytoplasm in retinal pigmented epithelial cells.^25^ While A2AR agonism led to increased cytoplasmic localization of p16 in TC28a2 chondrocytes, the precise cytoplasmic location was difficult to precisely identify and requires further research. Perhaps A2AR stimulation, which enhances chondrocyte autophagic flux, also promotes a p16 nuclear egress and autophagosomal incorporation akin to what has been illustrated in retinal pigmented epithelial cells. As for p21 localization, phospho‐p21 can translocate into the cytoplasm where it is anti‐senescent in contrast to its role in the nucleus.^24^ We previously found that A2AR stimulation increases autophagy and mitophagy in chondrocytes^6^, ^9^ and that A2AR activation leads to an increase in active phospho‐AKT within 30 min in both chondrocytes and osteoblasts,^5^, ^40^ which may explain the mechanism for A2AR‐regulated cytoplasmic localization of p16 and p21, respectively.

In addition to mitochondrial dysfunction and cellular senescence, altered sensing of cellular energy levels is a critical contributor to aging. We therefore assessed the effect of A2AR activation on the anti‐senescent interconnected energy‐sensing mediators Sirt1 and activated AMPK. Sirt1 and AMPK can activate each other (via LKB1 and elevated [NAD+], respectively) and are both upstream regulators of FoxO1/3, transcriptional regulators activated by A2AR ligation and required for normal cartilage development and maintenance.^9^, ^41^ Prior research has shown that protein kinase A (PKA), which is activated downstream of A2AR ligation, activates Sirt1, potentially explaining the early nuclear accumulation of nuclear Sirt1 followed by a second peak of nuclear Sirt1 that could result from the positive regulation of Sirt1 by AMPK.^42^, ^43^ LKB1, which is activated by Sirt1 deacetylation as well as via PKA phosphorylation, can shuttle from the nucleus to the cytoplasm to activate AMPK.^44^, ^45^ Both AMPK and Sirt1, which are thought to be mediators of longevity, enhance mitochondrial function and fatty acid oxidation, as we have previously reported for the effect of A2AR stimulation on chondrocyte mitochondria.^6^ Moreover, while high levels of reactive oxygen species (ROS) are detrimental to chondrocyte function, lower endogenous levels of ROS that are produced by healthy mitochondria are important signaling molecules. This balance between maintenance of mitochondrial function and ROS levels is essential in mitochondrial‐nuclear crosstalk that involves the pro‐longevity mitochondrial unfolded protein response (mtUPR) by increased expression of ATF4, ATF5, and CHOP (all significantly decreased in A2ARKO mouse chondrocytes) as well as the redirection of nuclear FoxO3 to the mitochondria where it interacts with mitochondrial Sirt3 to maintain cellular ROS homeostasis.^46^, ^47^ Hence, A2AR signaling may be anti‐senescence through its mito‐protective functions and mito‐nuclear crosstalk. This may allow lower levels of ROS to function as signaling molecules while avoiding excessive ROS production and oxidative damage. Indeed, there does appear to be a connection between ROS and p21 and p16 localization as discussed above. Taken together, A2AR activation may promote chondrocyte rejuvenation through three key hallmarks of aging: senescence, mitochondrial dysfunction, and deregulated nutrient‐sensing.^48^

Sirt1 is known to reduce senescence in OA cartilage and degenerative intervertebral disc cartilage through negative regulation of the p53‐p21 pathway as it can deacetylate the C‐terminal lysine K382 of p53, leading to its degradation by ubiquitin ligase MDM2 and thus reduced p21 protein levels.^21^, ^22^ In our previous research, we noted that p53 decreases significantly in the presence of CGS21680. With an A2AR‐mediated increase in Sirt1 and decrease in p53, we attempted to evaluate this connection by assessing K382Ac‐p53 levels. To our surprise, while the levels of full‐length K382Ac‐p53 did decrease as before, there was significantly more mRNA and protein for the anti‐senescent, pro‐longevity Δ133p53α variant (using anti‐K382Ac p53 and other antibodies that recognize all p53 isoforms), another mechanism by which A2AR stimulation prevents cellular senescence.

The demonstration that the anti‐senescent Δ133p53α variant is upregulated by A2AR stimulation is of particular interest considering our previous finding that A2AR stimulation promotes regeneration of cartilage in two animal models of OA. To our knowledge, this is the first report demonstrating a role for the Δ133p53α p53 variant in chondrocytes. This variant, transcribed in a FLp53‐dependent manner, is already known to display anti‐senescent effects in cells implicated in other aging‐associated degenerative conditions and improves the in vitro ability to reprogram differentiated cells. It significantly attenuates the senescent phenotype in fibroblasts derived from the prototypical premature aging disorder Hutchinson‐Gilford Progeria Syndrome (HGPS).^49^ Endogenous Δ133p53α in fibroblasts increases their reprogramming capacity into induced pluripotent stem cells (iPSCs) via specific dominant negative reduction of FLp53‐mediated expression of its target senescence genes (such as p21 and miR‐34a) without repression of FLp53 apoptotic and DNA repair functions.^50^ Astrocytes also express Δ133p53α, which functions in supporting proper CNS neuron function, and decreased levels can be observed in astrocytes in neurodegenerative disease like Alzheimer's disease (AD) and Amyotrophic Lateral Sclerosis (ALS) as a result of increased senescence.^51^ Δ133p53 also improves aging‐associated decreases in CD8 T‐cell immunity against viral infections and in tumor immunity.^52^

OA is a chronic disease and its pathology cannot be fully recapitulated by acute experimental manipulations, however, the data presented here does highlight the importance of the A2AR signaling pathway with respect to chondrocyte senescence in vitro and in vivo. A2AR activation reduces cellular senescence in chondrocytes in vitro by reducing p53 signaling and enhancing formation of its anti‐senescence variant Δ133p53α. Moreover, both in vitro and in vivo studies demonstrated A2AR ligation led to activation of AMPK and Sirt1 and altered p16 and p21 localization with a relatively reduced nuclear to cytoplasmic proportion of p16 and p21. Taken together these findings demonstrate that A2AR agonism is potentially an effective cartilage‐regenerative strategy for OA as both endogenous and exogenous stimulation of A2AR reduces chondrocyte senescence that occurs with cartilage deterioration in OA. This A2AR‐mediated effect to reduce chondrocyte senescence may underlie the previously observed cartilage regenerative phenotypic changes observed in animals treated with intra‐articular injections of the liposomally attached A2AR agonist.

## AUTHOR CONTRIBUTIONS

Benjamin Friedman designed the experiments, performed most of the data analysis, and wrote the manuscript. Carmen Corciulo provided data for parts of figures and aided in experimental analysis and decision‐making. Cristina M. Castro provided important background information and helped perform some of the experiments. Bruce N. Cronstein was the principal investigator/mentor for the project and was involved in the design and analysis of these studies. All authors reviewed the manuscript.

## DISCLOSURES

C. Corciulo and BN Cronstein have a patent for the methods and compositions for treating osteoarthritis and promoting cartilage formation (US Patent 10441541) assigned to NYU School of Medicine, and they are cofounders of Regenosine Inc. B Friedman, A Larranaga‐Vera, CM Castro & P Rabbani declare no competing financial interests.

## Supporting information


Data S1

## Data Availability

The data that support the findings of this study are available in the methods and/or supplementary material of this article.
