# Supplementary material for: Adenosine A2A receptor activation reduces chondrocyte senescence
Source: FASEB J. 2023 Mar 8;37(4):e22838. doi: 10.1096/fj.202201212RR (PMC11977601; doi:10.1096/fj.202201212RR)
Supplement: Supplementary file 1 — Data S1 [file FSB2-37-e22838-s001.docx]

**SUPPLEMENTAL FIGURES:**

**Supplemental Figure 1**

**
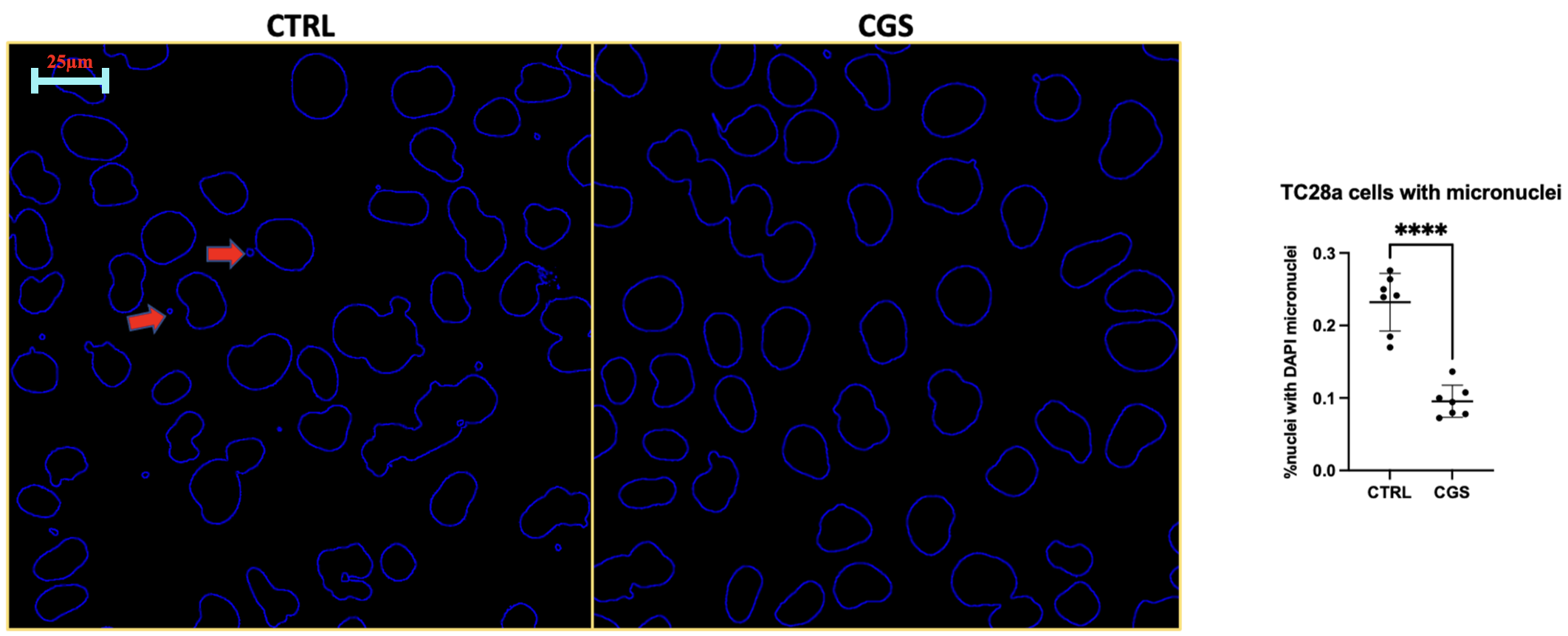
**

**Supplemental Figure 1.** DAPI visualization of TC28a2 of control and CGS21680 treated chondrocytes with nuclear and micronuclear edges that were enhanced via convolve filter with imageJ demonstrated to relative increase in number of micronuclei per 40x hpf as assessed in seven separate experiments. The red arrows point to micronuclei in proximity to their parent nucleus.

**Supplemental Figure 2**

**
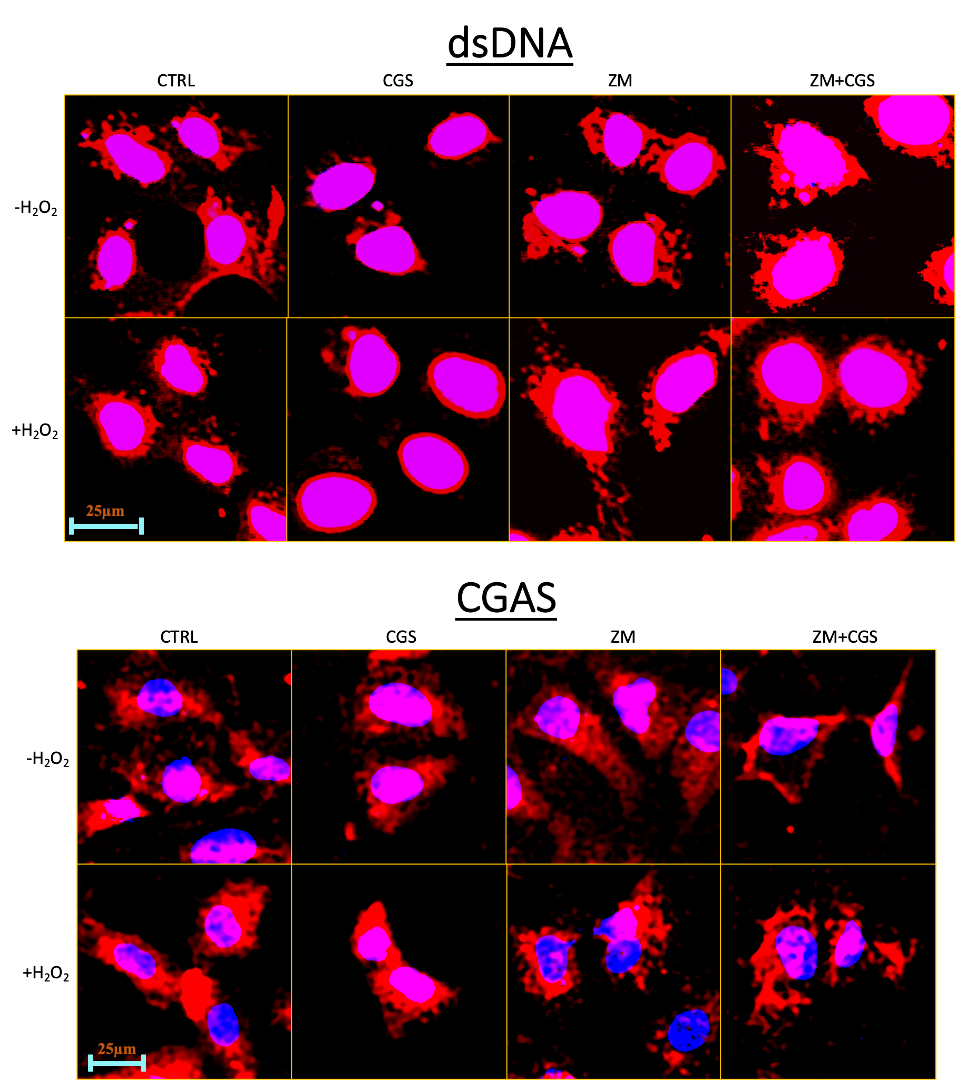
**

**Supplemental Figure 2.** Initial IF experiments demonstrate TC28a2 cells treated ±1µM CGS21680, ±1µM ZM241385, or both (pre-treated with ZM241385 for 5 minutes) for approximately 2 hours ±100µM H_2_O_2_ and stained by IF with either anti-dsDNA (top panel) or dual-function DNA sensor CGAS [1].

**Supplemental Figure 3**

**
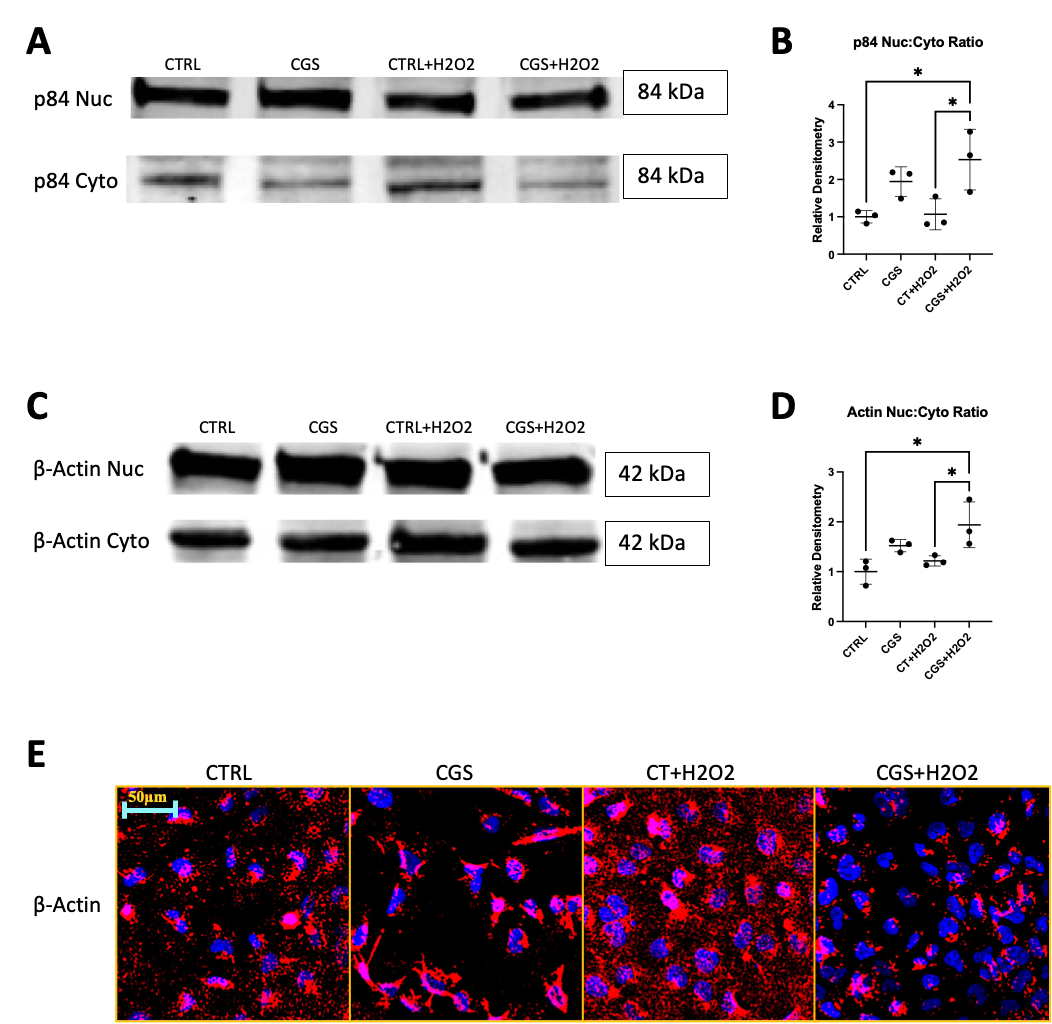
**

**Supplemental Figure 3. A2AR agonisms reduces cytoplasmic localization of nuclear marker protein p84 and increases the amount of beta-actin associated with the nuclear fraction in CGS21680 treated TC28a2 chondrocytes. (A)** and **(C)** show western blot of nuclear and cytoplasmic localization of p84 and actin, respectively, in cells treated ±CGS21680 and ±100µM H_2_O_2_. **(B)** and **(D)** show the graphical nuclear/cytoplasmic levels in each group in 3 experiments using a 1-way ANOVA calculation for significance for p84 and beta-actin, respectively. **(E)** IF analysis demonstrating TC28a2 cells with red IF immunostaining for beta-actin that shows the increase in CGS21680-mediated nuclear-associated actin.

**Supplemental Figure 4**

**
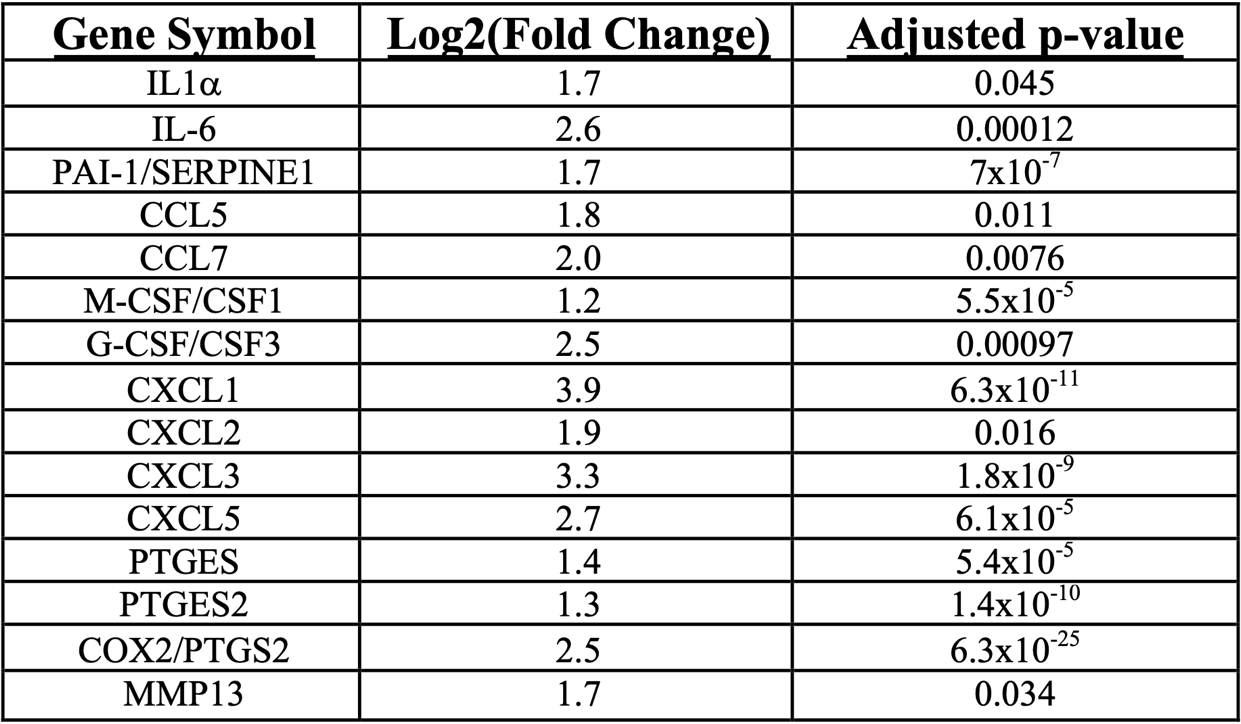
**

**Supplemental Figure 4.** **Analysis of chondrocyte gene expression changes comparing A2AR knockout chondrocytes to wildtype chondrocytes demonstrates predominant increase in major SASP-associated mediators.** Refer to the original publication for details: Castro, C. M. *et al.* Adenosine A2A receptor null chondrocyte transcriptome resembles that of human osteoarthritic chondrocytes. *Purinergic Signal* (2021). https://doi.org:10.1007/s11302-021-09788-5.

**Supplemental Figure 5**

**
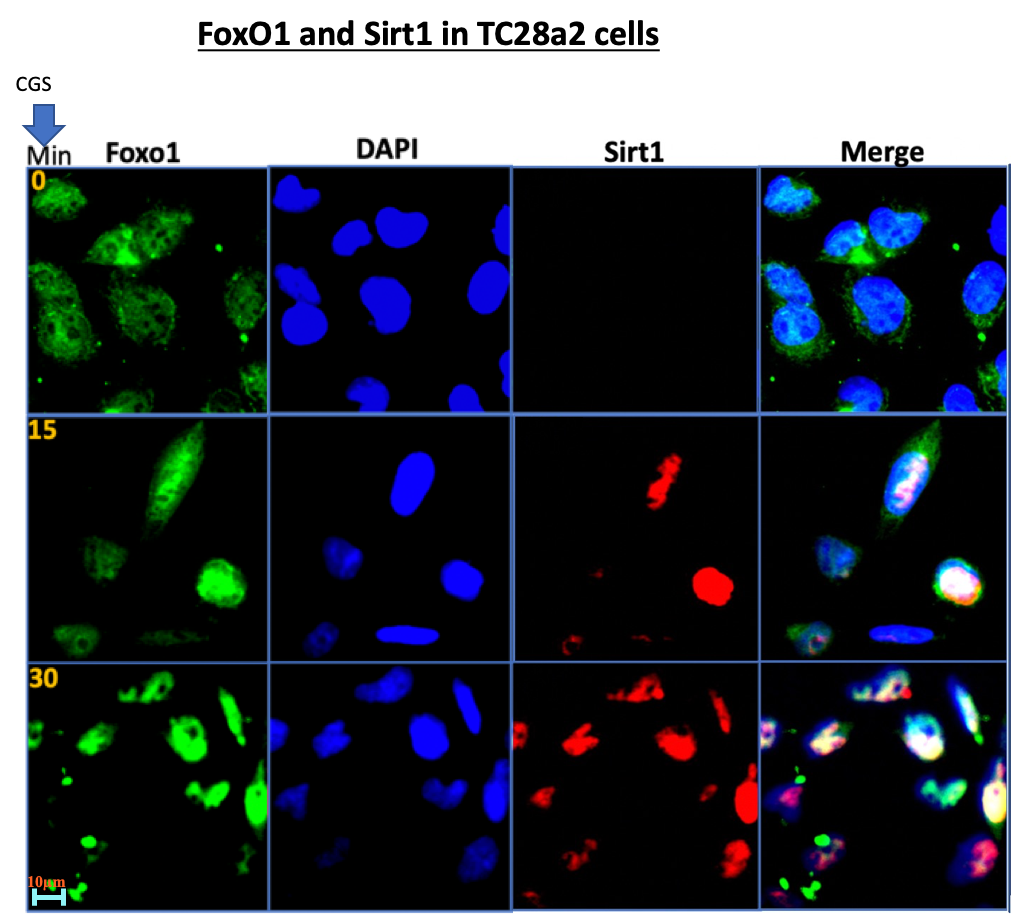
**

**Supplemental Figure 5.** **A2AR ligation leads to nuclear colocalization of Sirt1 and FoxO1.** TC28a2 cells were treated with 1µM CGS21680 and IF images over 30 minutes after treatment show FoxO1 (green), Sirt1 (red), and DAPI co-localization during this time.

**Supplemental Figure 6**

**
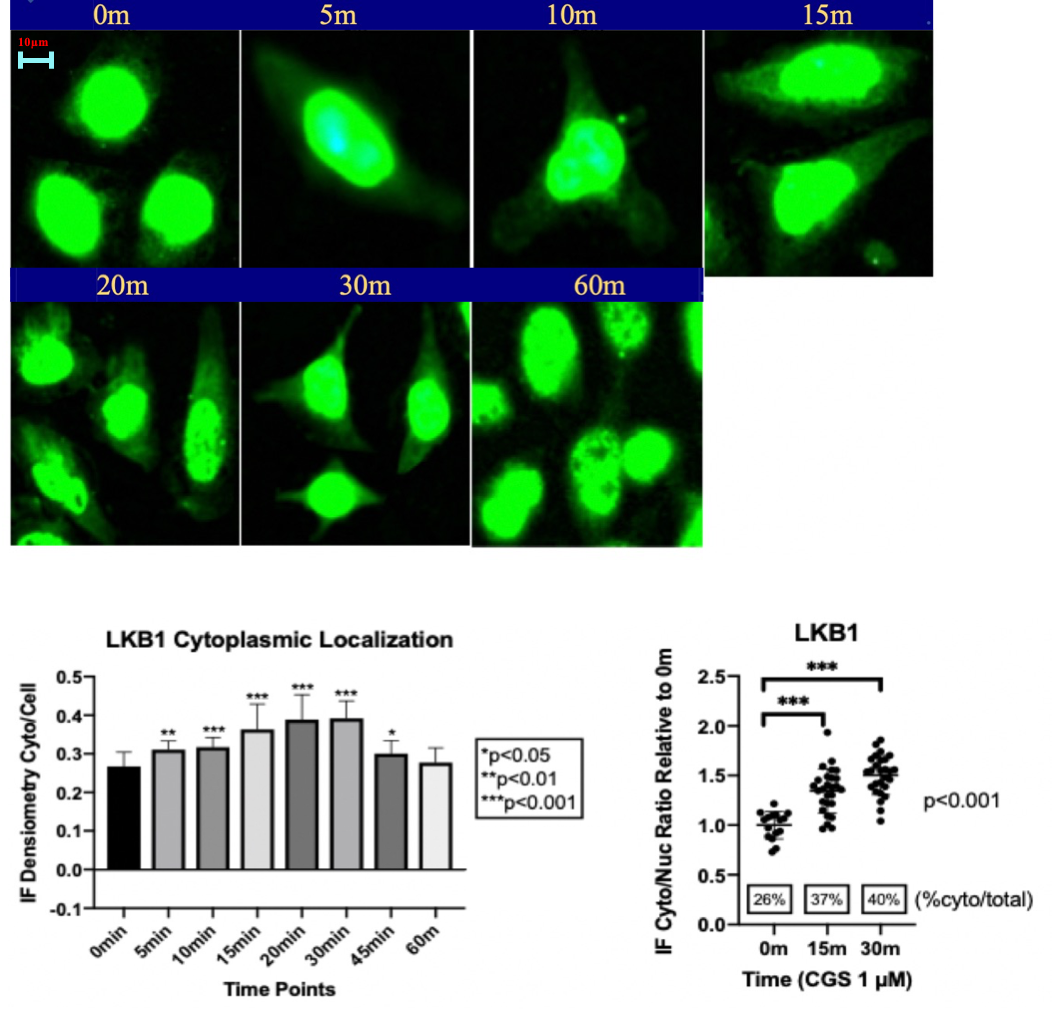
**

**Supplemental Figure 6. LKB1, upstream kinase for AMPK (cytoplasmic), is initially sequestered in the nucleus but translocates to the cytoplasm upon A2AR receptor stimulation.** IF analysis and evaluation of the compartmental fluorescence in numerous 40x HPFs in 3 different experiments at the time points indicated demonstrate that LKB1 can enter the cytoplasm beginning at 5 minutes until peak AMPK activation, which occurs in the cytoplasm. It subsequently becomes a predominantly nuclear protein at 60 minutes.

**Supplemental Figure 7**

**Supplemental Figure 7. Figure of p53 domains with antibodies employed to analyze for the presence of p53 and variants.**

**Supplemental Figure 8**

**
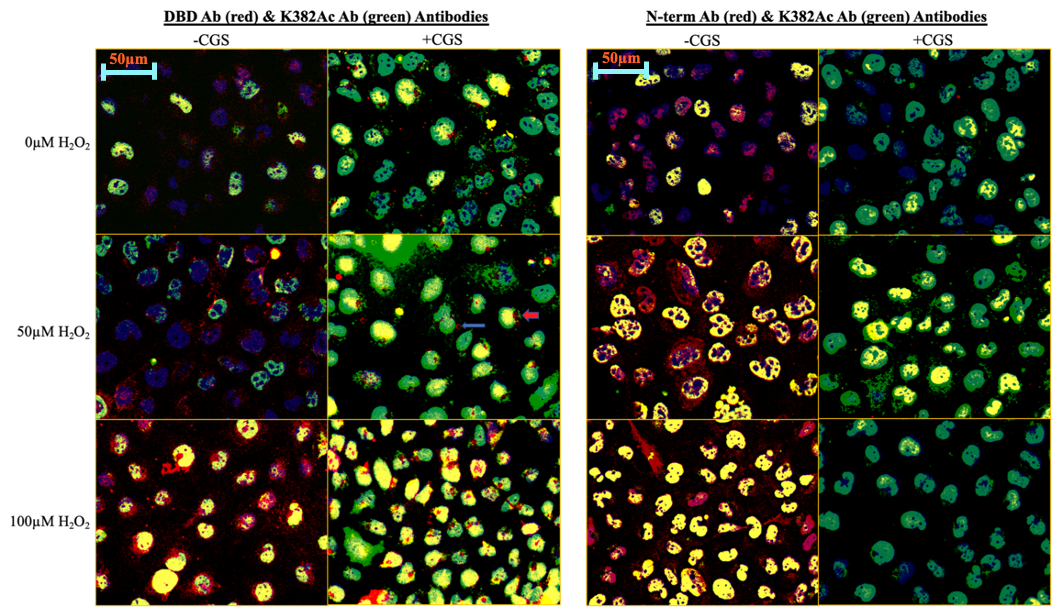
**

**Supplemental Figure 8. A2AR ligation reduces cytoplasmic p53 and leads to formation of vesicular structures exiting the nucleus.** The left panel shows co-localization of the p53 antibody that binds to the center of the DNA-binding domain (red) with the K382Ac p53 antibody (green); overlap (yellow). The right panel shows co-localization of the p53 antibody that binds to the N-terminus (red) with the K382Ac p53 antibody (green); overlap (yellow). Note on the left panel (50µM H2O2, +CGS21680), the presence of vesicular appearing deacetylated p53 (blue arrow) breaking through the nuclear envelope (red arrow). These were not observed in the right panel, indicating these structures may contain the deacetylated N-terminally truncated isoform.
